# Genome-Wide Identification of *Schistosoma japonicum* MicroRNAs Using a Deep-Sequencing Approach

**DOI:** 10.1371/journal.pone.0008206

**Published:** 2009-12-08

**Authors:** Jian Huang, Pei Hao, Hui Chen, Wei Hu, Qing Yan, Feng Liu, Ze-Guang Han

**Affiliations:** 1 Shanghai-MOST Key Laboratory for Disease and Health Genomics, Chinese National Human Genome Center at Shanghai, Shanghai, China; 2 Shanghai Institutes for Biological Sciences, Chinese Academy of Sciences, Shanghai, China; 3 National Institute of Parasitic Diseases, Chinese Center for Disease Control and Prevention, Shanghai, China; Sabin Vaccine Institute, United States of America

## Abstract

**Background:**

Human schistosomiasis is one of the most prevalent and serious parasitic diseases worldwide. *Schistosoma japonicum* is one of important pathogens of this disease. MicroRNAs (miRNAs) are a large group of non-coding RNAs that play important roles in regulating gene expression and protein translation in animals. Genome-wide identification of miRNAs in a given organism is a critical step to facilitating our understanding of genome organization, genome biology, evolution, and posttranscriptional regulation.

**Methodology/Principal Findings:**

We sequenced two small RNA libraries prepared from different stages of the life cycle of *S. japonicum*, immature schistosomula and mature pairing adults, through a deep DNA sequencing approach, which yielded ∼12 million high-quality short sequence reads containing a total of ∼2 million non-redundant tags. Based on a bioinformatics pipeline, we identified 176 new *S. japonicum* miRNAs, of which some exhibited a differential pattern of expression between the two stages. Although 21 *S. japonicum* miRNAs are orthologs of known miRNAs within the metazoans, some nucleotides at many positions of *Schistosoma* miRNAs, such as miR-8, let-7, miR-10, miR-31, miR-92, miR-124, and miR-125, are indeed significantly distinct from other bilaterian orthologs. In addition, both miR-71 and some miR-2 family members in tandem are found to be clustered in a reversal direction model on two genomic loci, and two pairs of novel *S. japonicum* miRNAs were derived from sense and antisense DNA strands at the same genomic loci.

**Conclusions/Significance:**

The collection of *S. japonicum* miRNAs could be used as a new platform to study the genomic structure, gene regulation and networks, evolutionary processes, development, and host-parasite interactions. Some *S. japonicum* miRNAs and their clusters could represent the ancestral forms of the conserved orthologues and a model for the genesis of novel miRNAs.

## Introduction

Schistosomes are members of the phylum Platyhelminthes. However, unlike most other platyhelminths, dioecious schistosomes can cause severe human schistosomiasis, which remains one of the most prevalent and serious parasitic diseases worldwide [1]. They are highly adapted for life inside their mammalian hosts, where they can survive for years [2]. Although genomic information has improved our understanding of the biology of schistosomes and their interactions with hosts [3], increasing our knowledge of microRNAs (miRNAs) may reveal unique classes of riboregulators that shape evolutionary characteristics throughout different animal phyla [4], uncover developmental genetic switches [5], and develop novel biomarkers for the parasitic disease [6], [7].

MicroRNAs (∼22 nt in length) play gene-regulatory roles in numerous eukaryotic lineages, including plants, animals and fungi. Identification of comprehensive sets of miRNAs and other small regulatory RNAs in different organisms is a critical step to facilitate our understanding of genome organization, genome biology and evolution [8]. RNA interference (RNAi) has previously been described in *Schistosoma mansoni* and *S. japonicum*, for which the addition of exogenous dsRNA resulted in a measurable suppression of target gene expression [9]–[12]. This suggests that schistosomes possess the molecular machinery that contains an effector nuclease complex, known as the RNA-induced silencing complex (RISC), which recognizes and destroys homologous target mRNAs in an endonucleolytic manner [13], [14]. Recently, a Dicer-1 like (EF204544) multi-domain nuclease that is responsible for cutting double-strand RNAs into short interfering RNAs (siRNAs) approximately 21 nucleotides (nt) long, and argonaute (Ago) effector proteins that target mRNA molecules for silencing or destruction under guidance by miRNAs, were identified in *S. mansoni* [15], [16]. This implies that miRNAs could be generated by action of Dicer and play important roles in post-transcriptional regulation. Actually, few miRNAs were recently identified from *S. japonicum* [17]. However, the identification of numerous schistosome miRNAs should be further performed for characterizing evolutionary position of schistosomes spanning the ecdysozoans and deuterostomes, gene regulation during development, and host-parasite interplay of the blood flukes.

## Methods

### Preparation of schistosome specimens

Preparation of schistosome specimens was conducted as previously described [3], [18]. Briefly, cercariae of *S. japonicum* were shed from naturally infected *Oncomelania hupensis* snails collected from fields in Anhui Province of China. Each rabbit was experimentally infected percutaneously with 1,000 cercariae. Hepatic schistosomula and adult worms were obtained from the mesenteric veins and liver of infected rabbits at 2 weeks and 6–7 weeks after infection, respectively. Mixed-sex adults and hepatic schistosomula were then washed thoroughly in PBS to remove host cell debris, and stored at −80°C for further analysis. All animals were handled in strict accordance with the guidelines defined by the relevant national and/or local animal welfare bodies, and all animal experiments were approved by the ethics committee of the Chinese National Human Genome Center (Shanghai, China).

### RNA extraction, construction of small RNA libraries, and DNA sequencing

The worms were dissected and homogenized in the lysis buffer. Total RNA was then extracted with TRIzol (Invitrogen, Carlsbad, CA) according to the manufacturer's instructions. RNA concentration and purity were evaluated photometrically by measuring the absorbance at 260 nm and 280 nm, through NanoDrop ND-1000 spectrophotometer (Nanodrop Technologies, Wilmington, DE) and Agilent 2100 Bioanalyzer (Agilent Technologies, Palo Alto, CA). RNA samples were stored at −80°C.

For small RNA library construction and deep sequencing, the 18–30 nt size range of RNA was enriched by polyacrylamide gel electrophoresis (PAGE) and then 20 µg of the purified small RNA from each developmental stage was subject to DNA sequencing with an Illumina Genome Analyzer (Illumina, San Diego, CA), according to the manufacturer's instructions. In brief, proprietary adapters were then ligated to the 5′ and 3′ termini of these small RNAs, which the ligated small RNAs were then used as templates for cDNA synthesis. The cDNA was amplified with 18 PCR cycles to produce libraries that were sequenced using Solexa's proprietary sequencing-by-synthesis method. DNA sequencing was performed with an Illumina Genome Analyzer. The image files generated by the sequencer were then processed to produce digital-quality data. In this study, 35 nt small RNA reads (*.fq) were produced by BGI (Beijing Genome Institute at Shenzhen) using a Solexa/Illumina sequencer. This raw dataset was processed with a bioinformatics' pipeline as follows: (1) remove low quality reads; (2) trim 3′ prime adaptor sequences by a modified dynamic programming algorithm; (3) remove adaptor contaminants formed by adaptor and adaptor ligation; (4) collect short RNAs ranged from 18–30 nt and draw size distribution; (5) remove those sequences with ployA tail. And then we got the clean reads of full-length small RNA sequences for further analysis.

### Computational analyses

After low quality sequence reads were removed according to the criteria of Solexa/Illumina, the identical sequence reads were tabulated to produce a ‘read count’ score. Duplicated sequences were eliminated from the initial dataset to produce a non-redundant set of unique sequences, hereafter referred to as sequence tags. After trimming the ligated adaptor sequences, identical sequences were counted as their expression abundances.

To determine whether these small RNA sequences from *S. japonicum* are considered as candidate miRNAs, these cleaned small RNA sequences described above were mapped to the draft *S. japonicum* genome sequences (sjr2_contig.fasta) using SOAP (Short Oligonucleotide Alignment Program) (http://soap.genomics.org.cn). Only those mapped perfectly onto the draft genome were further considered as candidate miRNAs.

To identify potential miRNA genes, the MIREAP algorithm (http://sourceforge.net/projects/mireap) was employed to obtain all candidate precursors with hairpin-like structures that were perfectly mapped by sequencing tags. In brief, two putative miRNA precursor sequences (one encompassing 10 nt upstream and 70 nt downstream, assuming the miRNA locates to the 5′-prime arm of the RNA hairpin; the other encompassing 70 nt upstream and 10 nt downstream, assuming the miRNA locates to the 3′-prime arm of the hairpin) were folded using RNAfold [19]. The pairing numbers of the mature sequence within a given RNA hairpin should not be less than 14 nt, while the maximal bulge should be less than 4 nt and the asymmetry between miRNA and miRNA star (miRNA*) should be less than 5 nt. Because the miRNA and miRNA* duplexes are products of Drosha and Dicer processing, a 3′-terminus overhang of two nucleotide bases over the 5′-terminus was requested.

For homology analysis, these predicted mature *S. japonicum* miRNAs were compared with known miRNAs from other organisms (http://www.mirbase.org/). If both a given *S. japonicum* miRNA and known miRNAs reciprocally share the highest homology with more than 80% identical nucleotides, including the same seed sequences (2–7 nt), the *S. japonicum* miRNA was considered as a ortholog and thus named after the known miRNA. In this work, we also employed these *S. japonicum* miRNAs to predict the orthologs of *S. mansoni*, according to the same criteria, through search against draft *S. mansoni* genome. Moreover, we extracted 2–7 nt of these *S. japonicum* miRNAs as the potential “seed sequences” to search against all published known miRNAs from other organisms. When a “seed sequence” was perfectly matched with the sequence at the same location of a known miRNA, the *S. japonicum* miRNA was considered to be homologous with the known miRNA.

### MiRNA target prediction

We first predicted the 3′-untranslational regions (UTRs) by integrating the predicted mRNAs (http://lifecenter.sgst.cn/schistosoma/en/schistosomaCnIndexPage.do) and the released EST annotation (http://192.168.13.13/sj-proteome/download.htm) [3], [18] for *S. japonicum*, and then employed the miRanda program (http://www.microrna.org/microrna/getDownloads.do) to predict the target genes for the 176 *S. japonicum* miRNAs [20]. The parameters used in miRanda were a gap opening penalty of −8; a gap extension penalty of −2; a score threshold of 50; an energy threshold of −20 kcal/mol; a scaling parameter of 2.

## Results

### Screening of potential *S. japonicum* miRNAs

Increased knowledge of schistosome microRNAs may provide further information about the unique classes of riboregulators. We first conducted a search for potential homologues of the genes involved in miRNA mechanisms against the draft *S. japonicum* genome accordingly. In addition to Dicer-1 (Sjc_0069770) and three Ago genes (Sjc_0044720, Sjc_0045200 and Sjc_0103990): we found that the *S. japonicum* genome could encode the homologues of Drosha (Sjc_0048900) and Pasha (Sjc_0013270) (Table S1), which could recognize the miRNA primary transcript and cleave it to create the pre-miRNA hairpin [21]. This result supported the hypothesis that *S. japonicum* is capable of generating endogenous miRNAs that carry out post-transcriptional regulation.

**Figure 1:**
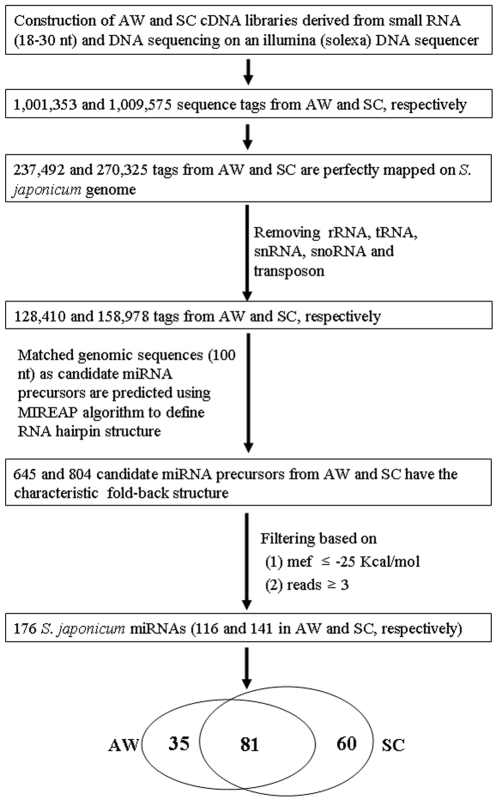
The flowchart of screening and identifying *S. japonicum* miRNAs. AW, mixed adult worms; SC, hepatic schistosomula.

**Figure 2:**
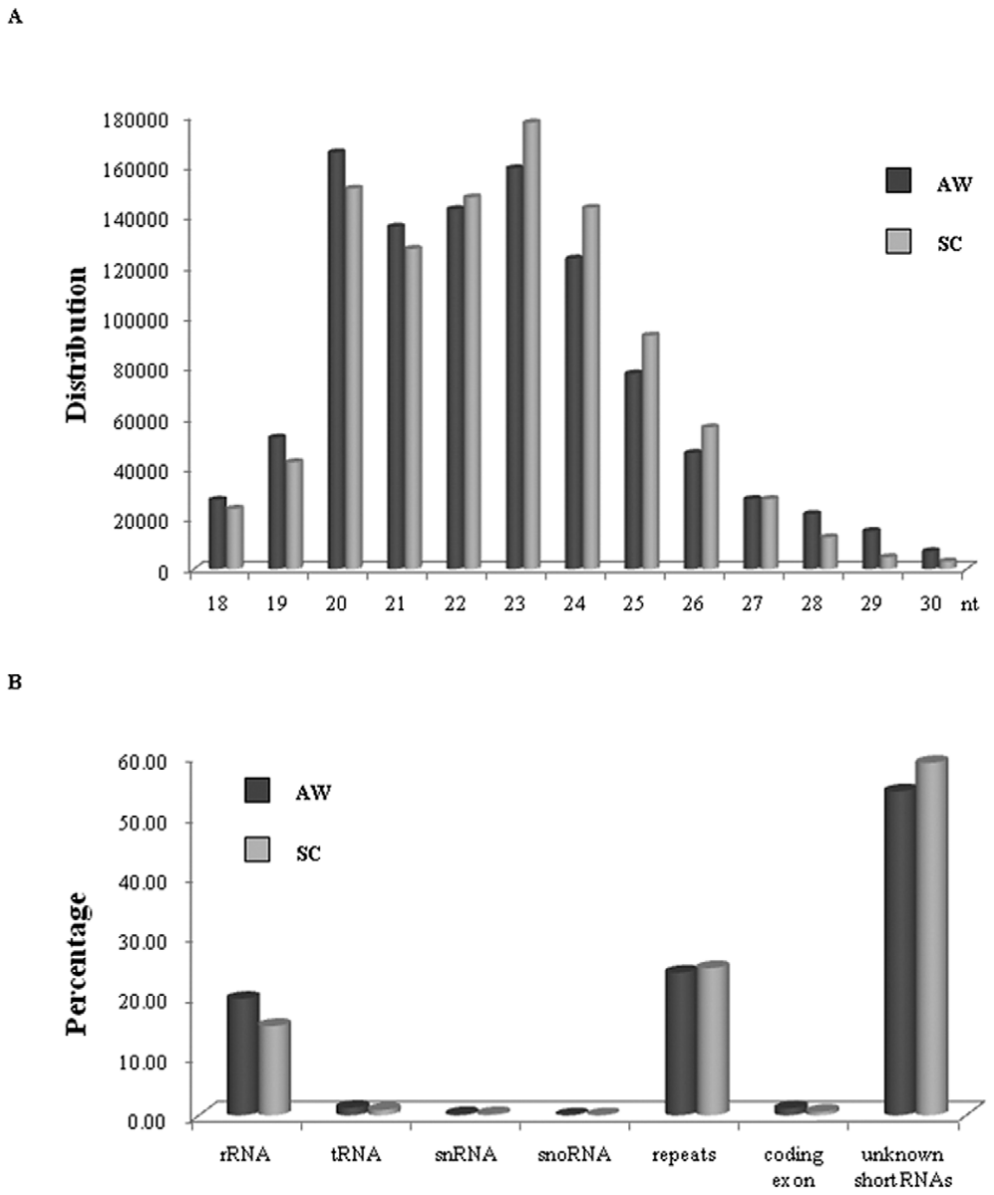
Size and frequency distribution of the sequencing reads from both cDNA libraries as well as the classification of small RNAs. (A) Length distribution of the non-redundant sequencing reads. (B) Classification of the sequenced small RNA tags from adult worms (AW) and immature hepatic schistosomula (SC), respectively.

To survey *S. japonicum* microRNAs and further understand their biological function, we constructed complementary DNA libraries derived from 18–30 nt RNAs isolated from both schistosomula (SC), the immature unpaired worms, and mature pairing adults (AW), respectively (Figure 1), where the pairing of schistosomes are essential for their maturation and egg-laying. We then sequenced them using an Illumina (Solexa) DNA sequencer, which yielded ∼12 million raw reads containing a total of ∼2 million non-redundant tags with high-quality reads (1,001,353 and 1,009,575 sequence tags generated from AW and SC, respectively). The sequencing tags were merged and the expression level of each unique tag was normalized to transcripts per million (TPM) as previously described [22]–[25]. Our results showed that the majority of these small RNAs were between 20 to 24 nt in length (Figure 2A).

To determine whether these small RNA sequences from *S. japonicum* are considered as candidate miRNAs, these small RNA sequences described above were mapped to the draft *S. japonicum* genome sequences (sjr2_contig.fasta) (http://lifecenter.sgst.cn/schistosoma/en/schistosomaCnIndexPage.do) using SOAP. The result indicated that 237,492 and 270,325 non-redundant sequence tags from AW and SC were perfectly mapped onto the *S. japonicum* genome, respectively (Figure 1). Then, we further excluded these small RNAs that matched with known rRNAs, tRNAs, small nuclear RNAs (snRNAs) and small nucleolar RNAs (snoRNAs) deposited in the Rfam database (ftp://selab.janelia.org/pub/Rfam/) and NCBI GenBank (http://www.ncbi.nlm.nih.gov/GenBank). We also masked the repeat sequences using Exonhunter (http://lifecenter.sgst.cn/schistosoma/en/schistosomaCnIndexPage.do) and RepeatMasker programs [26] (http://www.repeatmasker.org), and ones mapped to protein-coding exons. The data indicated that about 16–21% of the small RNAs matched with rRNAs, tRNAs, snRNAs and snoRNAs (Figure 2C and 2D), ∼24% mapped to repeat sequences, including transposons and retrotransposons, and ∼1.2% mapped to protein-coding exons. After removing the above small RNAs, a total of 128,410 and 158,978 tags from AW and SC are further analyzed for the identification of *S. japonicum* miRNAs (Table S2).

To identify potential miRNA genes, the MIREAP algorithm [19] was then employed to obtain all candidate precursors with hairpin-like structures that were perfectly mapped by sequencing tags (see Methods). These tags were filtered by the following criteria: (1) The minimum free energy (mfe) should be less than <−25 Kcal/mol; (2) at least 3 Solexa sequencing reads demonstrating the same 5′-terminus. The results showed that every candidate precursor of 645 and 804 sequence tags from AW and SC possesses a characteristic RNA hairpin structure, respectively (Figure 1). Hence, these sequence tags were considered as potential miRNAs to be further analyzed.

### Identification of *S. japonicum* miRNAs

To further identify *S. japonicum* miRNAs, we considered the following properties that have proved useful for distinguishing bilaterian miRNAs from other types of small RNAs as the follow criteria: (1) the presence of reads mapped to an inferred RNA hairpin with pairing characteristics of known miRNA hairpins; (2) the seed sequence with similarity to known miRNAs from other species (miRBase 12.0) [27]; and (3) the RNA wasn't mapped to a genomic region with an annotation suggesting a non-miRNA biogenesis (Figure 1) [28]–[30]. Based on above criteria, a total of 176 *S. japonicum* miRNA genes were identified (Table S3) (http://omics.biosino.org:14000/kweb/sj_miRNA/index.html or http://function.chgc.sh.cn/sj_miRNA/).

**Figure 3:**
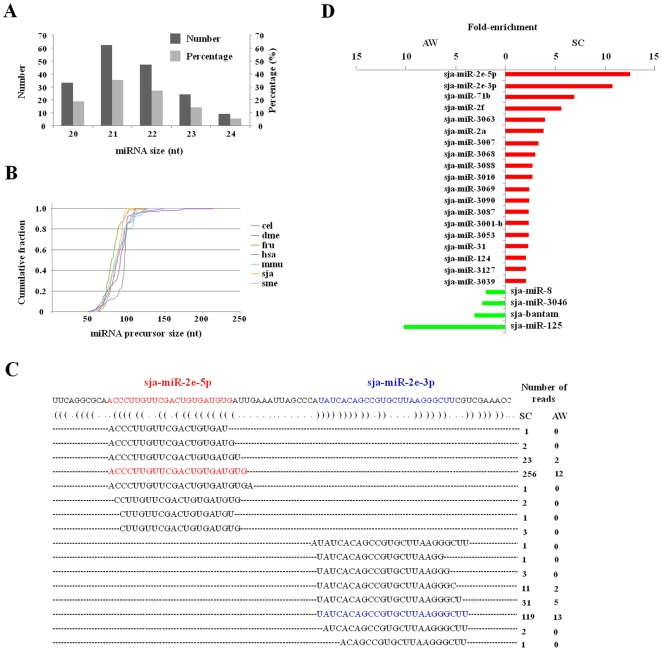
Identification and properties of *S. japonicum* miRNAs. (A) Length distribution of all identified *S. japonicum* miRNAs. The left and right Y-axes indicate the number and percentage of miRNAs, respectively. (B) Cumulative length distributions of miRNA precursors from *S. japonicum* and other bilaterian animals. The size distribution of *S. japonicum* miRNA precursors is similar to that of other bilaterian animals, including *C. elegans* (cel), *Schmidtea mediterranea* (sme), *D. melanogaster* (dme), *Fugu rubripes* (fru), mice (mmu), and humans (hsa). (C) The sequences and numbers of sequencing reads matching the sja-miR-2e hairpin. The sequence of the sja-miR-2e hairpin is displayed above the bracket-notation of its predicted secondary structure, as determined by RNAfold. Sequenced small RNAs from immature schistosomula (SC) and adult worms (AW) that map to the hairpin are aligned below, with the number of reads shown on the right. Both miR-2e-5p and the conserved miR-2e-3p were designated as reciprocal miRNA and miRNA* species and are indicated in red and in blue, respectively. (D) Relative expression levels of *S. japonicum* miRNAs, as indicated by fold enrichment through normalizing the frequency of sequencing reads from the AW and SC samples.

In addition to known *S. japonicum* miRNAs, sja-let-7, sja-miR-71a, sja-miR-125 and sja-bantam [17], the remaining 172 miRNAs were first recognized in *S. japonicum*. Moreover, our data showed that 141 (80%) of the 176 miRNAs were from the SC library, while 116 (66%) were from the AW library, and 81 (46%) of the miRNAs were found in both libraries. All mature miRNAs are between 20–24 nt long (Figure 3A), of which 62% were 21 or 22 nt. All *S. japonicum* miRNA precursors are 66–101 nt in length and demonstrated the typical RNA hairpin structure of RNA hairpins. The size distribution of these hairpins was similar to that of other bilaterian animals, including *C. elegans*, *Drosophila*, fish, mice, and humans (Figure 3B).

As known, those duplex reads correspond to an intermediate of miRNA biogenesis in which the miRNA and opposing segment of the hairpin, called the miRNA star (miRNA*), are excised from the hairpin through successive action of Drosha and Dicer RNase III endonucleases [28]–[30]. Sequencing reads from one arm of the RNA hairpin usually greatly exceeded those from the opposite arm, enabling unambiguous annotation of the duplex miRNA and the miRNA*. To explore the candidate miRNA* in *S. japonicum*, the potential miRNA sequences were aligned to the miRNA precursors. We found the presence of some reads from both arms of the hairpin, which formed a duplex with 2-nucleotide 3′-overhangs when paired to each other. Notably, a pair of miRNAs, the highly conserved miRNA-2e-3p and the newly identified miRNA-2e-5p, were derived from two arms of the same RNA hairpin precursor (Figure 3C).

To gain insight into possible mechanisms involving miRNAs across the development stages of *S. japonicum*, we compared the miRNA reads between the cDNA libraries of immature and mature worms. The result showed that some miRNA genes, including miRNA-2e-5p, miRNA-2e-3p, miR-71b, miR-2a, miR-2f, miR-124 and miR-31, tended to be enriched in immature worms, whereas miR-125, miR-8 and bantam exhibited a higher abundance in mature adults (Figure 3D), suggesting that different miRNAs could play a distinct role in different development stages.

### Genomic clusters of *S. japonicum* miRNAs

**Figure 4:**
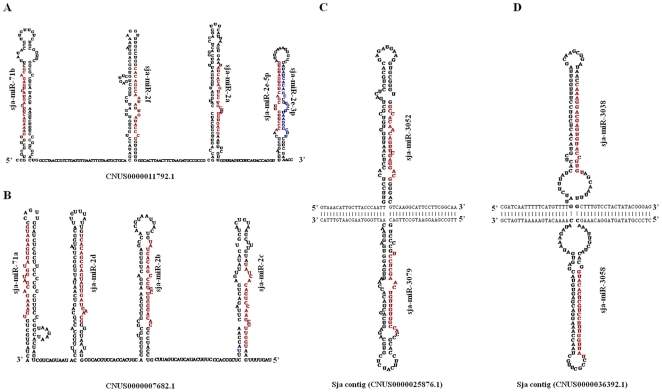
Genomic origin of *S. japonicum* miRNAs. (A) A miRNA cluster consisting of miR-71b, miR-2f, miR-2a miR-2e-5p, and miR-2e-3p in tandem on the same genomic contig (CNUS0000011792.1). (B) A similar miRNA cluster containing miR-71a, miR-2d, miR-2b, and miR-2c was mapped in an inverted orientation to another genomic contig (CNUS0000007682.1). (C,D) Two pairs of miRNAs, sja-miR-3052 and sja-miR-3079, as well as sja-miR-3038 and sja-miR-3058 were generated from sense and antisense DNA strands within the same genomic loci (CNUS0000025876.1 and CNUS0000036392.1), respectively.

A subset of miRNA genes are well-known to reside in local genomic clusters with possible operon-like organization [31]. To explore whether there are miRNA clusters in *S. japonicum* genome, all of the identified *S. japonicum* miRNAs were mapped to S. japonicum genome. Here only two miRNA clusters identified by this study exhibited the similar genomic structure and components consisting of miR-71 and miR-2 family members. Interestingly, these five highly expressed miRNAs, miR-71b, miR-2a, miRNA-2e-5p, miRNA-2e-3p and miR-2f, were clustered in tandem at the same genomic locus (CNUS0000011792.1) (Figure 4A), whilst other miR-71 and miR-2 family members, miR-71a, miR-2d, miR-2b, and miR-2c, were clustered at another genomic locus (CNUS0000007682.1) in an inverted orientation (Figure 4B).

In addition to these miRNA gene clusters, we also found that both sense and antisense DNA strands within the same genomic loci could generate the distinct miRNAs. For examples, the pairing miRNAs, including miR-3052 and miR-3079 as well as miR-3038 and miR-3058, were produced by different strands with typical RNA hairpin precursors located in the same genomic regions (CNUS0000025876.1 and CNUS0000036392.1), where the sense strands encode the miR-3052 and miR-3038 while antisense strands generate the miR-3079 and miR-3058 (Figure 4C and 4D), respectively. It is known that distinct miRNAs derived from both DNA strands of the same genomic loci could play crucial role in regulating the development by targeting distinct genes [32].

### Evolution of *S. japonicum* miRNA genes

**Figure 5:**
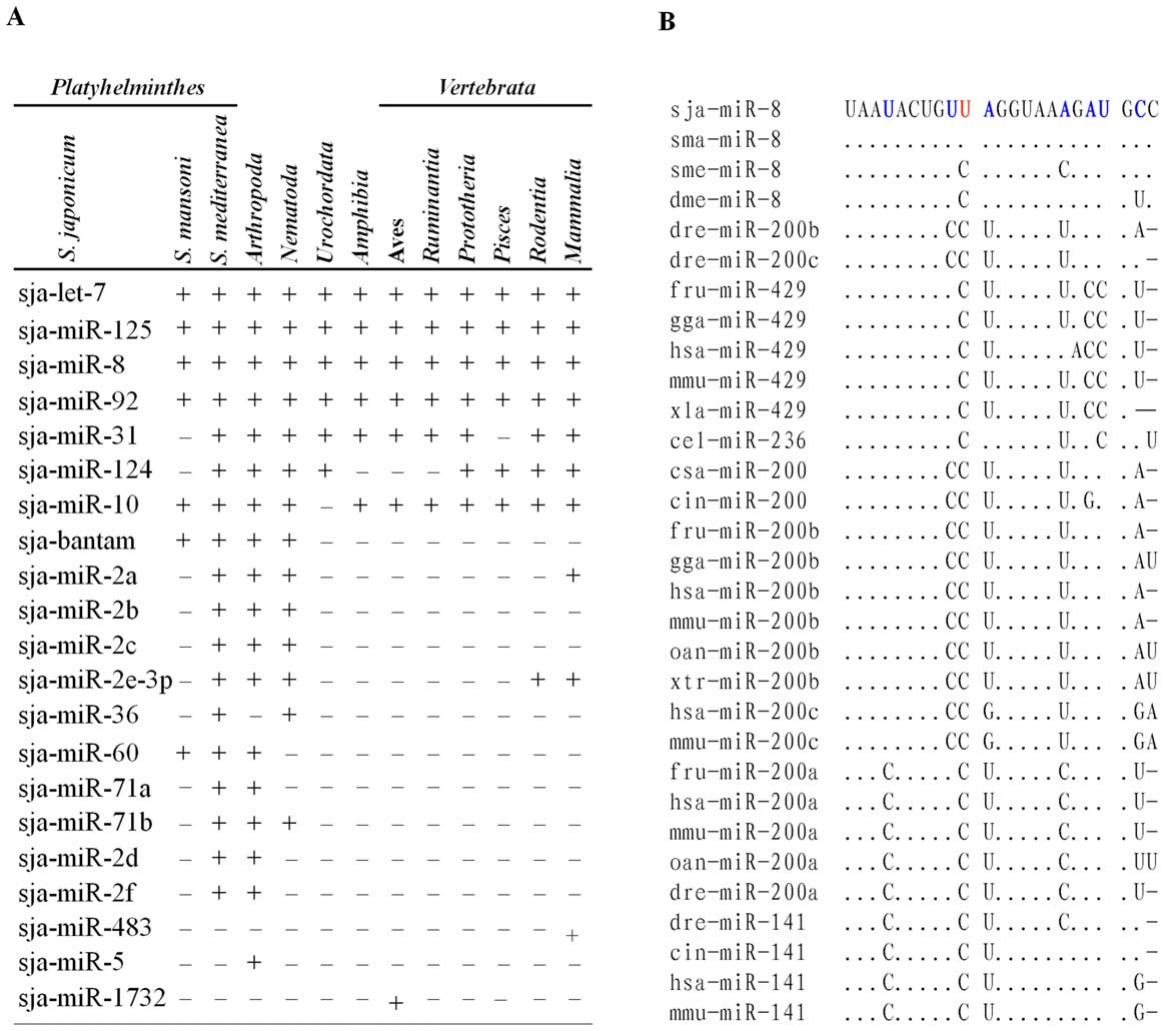
Evolutionary analysis of *S. japonicum* miRNAs. (A) 21 *S. japonicum* miRNAs are orthologs of known miRNAs from other bilaterian animals. “+” indicates the high homology with the orthologs of the indicated species or phylum of animals, while “−” indicates no homology with to the orthologs. (B) Alignment of miR-8 homologues from some bilaterian animals. The nucleotides in red represent substitution that is specific to schistosomes, while blue letters indicate nucleotide substitutions found in orthologs or their paralogs in some other species and black letters represent the conserved nucleotides. Abbreviations: sja, *S. japonicum*; sma, *S. mansoni*; sme, *S. mediterranea*; dme, *D. melanogaster*; dre, *D. rerio*; fru, *F. rubripes*; gga, *G. gallus*; hsa, *H. sapiens*; mmu, *M. musculus*; xla, *X. laevis*; cel, *C. elegans*; csa, *C. savignyi*; cin, *C. intestinalis*; oan, *O. anatinus*; xtr, *X. tropicalis*.

To further explore the evolutionary features of *S. japonicum* miRNAs, we compared the collection of miRNAs with published miRNAs from other metazoans. Our data showed that 160 (91%) of the 176 *S. japonicum* miRNAs share 90 different 5′-seed sequences (2–7 nt) with known miRNAs from other animals (Table S4 and Table S5), of which 21 are orthologs of known miRNAs within the metazoans (Figure 5A). It should be pointed out that *S. japonicum* miR-3068 shares the same seed (1–8 nt) with miR-2029 of *Nematostella vectensis* (sea anemone), one of the earliest branching animals in the Eumetazoa; and that *S. japonicum* miR-3040 possesses the same 2–9 nt as miR-2019 of *Amphimedon queenslandica* (sponge), one of the earliest branching animals in the Metazoa [4]. This implies that these miRNAs could be derived from ancient ancestral genes.

Here we also employed these *S. japonicum* miRNA precursors to interrogate against draft *S. mansoni* genome (http://www.genedb.org/genedb/smansoni/index.jsp) by homologous comparison. Those *S. manson*i genomic sequences with more than 95% homology to *S. japonicum* miRNA precursors were considered as candidate miRNA precursors. The *S. manson*i miRNAs were then predicted according to the same criteria described above. The result indicated that 31 potential *S. mansoni* miRNA precursors with typical RNA hairpins and mature forms, which are homologues of *S. japonicum* orthologs, were recognized (Table S6). Some of the common *Schistosoma* miRNAs, including let-7, miR-8, miR-10, miR-31, miR-92, miR-124 and miR-125, are evolutionally conserved across bilaterian animals (Figure 5A). We also noted that 15 *S. japonicum* miRNAs are orthologs of the published orthologs from *Schmidtea mediterranea* (Figure 5A) [33], a multicellular animal belonging to the phylum *Platyhelminthes*.

Moreover, schistosomes also share some known miRNAs, such as bantam, miR-36, miR-60, miR-2 and miR-71 with *Arthropoda* and/or *Nematoda* animals. Interestingly, *Schistosoma* miR-8, shared by both schistosomes, was homologous with *Arthropoda* orthologs, as well as *Nematoda* miR-236, *Urochordata* and *Vertebrata* miR-141, miR-200 and miR-429 (Figure 5B). The tenth nucleotide uracil (U) of *Schistosoma* miR-8 is significantly distinct from cytidine (C) of all orthologs or their paralogs, including *S. mediterranea* miR-8. miR-8 of *Platyhelminthes* (*Schistosoma* and *S. mediterranea*) and *Arthropoda* share the same eleventh adenine (A) with *Nematoda* miR-236, which is different from U or guanine (G) of the same position of *Urochordata* and *Vertebrata* miR-141, miR-200 and miR-429 (Figure 5B).

Phylogenetic analysis implying that *Schistosoma* miR-8, which exhibited a different clade from the *S. mediterranea* ortholog, could represent an ancient prototype of *Arthropoda* orthologs, and share the ancestral miRNA gene origin with *Urochordata* and *Vertebrata* miR-141 and miR-200a (Figure S1). Similarly, we also aligned other highly conserved miRNAs, such as let-7, miR-10, miR-31, miR-92, miR-124 and miR-125, across bilaterian animals using WebLogo tool. Some nucleotides at many positions of *Schistosoma* miRNAs are indeed significantly distinct from other bilaterian orthologs (Figure S2).

### Target gene prediction of *S. japonicum* miRNAs

Predictions of target genes are a very complex process and require experimentation. Although so, we attempt to explore the potential functions of these *S. japonicum* miRNAs, through predicting their potential target genes using miRanda software, an open-source algorithm [34], [35], via searching against the 3′-untranslational regions (UTR) of *S. japonicum* protein-coding genes. The data set was deduced by integrating the predicted mRNA with known important functions and published EST data [3], [18]. The result showed that each miRNA could regulate many target genes, from several to hundreds, and some development-associated genes could be regulated by many miRNAs (Table S7).

We investigate the relationship between the genomic loci of *S. japonicum* miRNAs and their host genes. We noted that 52 *S. japonicum* miRNAs were located within the intronic regions of some predicted protein-coding genes (Table S8), although the majority (70%) of these miRNAs was mapped to intergenic regions. *S. japonicum* miR-3148 was mapped to the sixth intron of the heterogeneous nuclear ribonucleoprotein K (hnRNP K) (Figure S3). Interestingly, the miR-3148 sequence is highly similar to the 3′-UTR of hnRNP K, implying that miR-3148 could regulate the stability or translation of hnRNP K mRNA.

## Discussion

Although the draft genomic information has provided a profound understanding of schistosome biology and their host-pathogen interactions [36], increased knowledge of schistosome microRNAs may provide further information about the unique classes of riboregulators that have helped shape evolutionary characteristics throughout animal phyla, uncover developmental genetic switches, and develop novel biomarkers for detection of the parasitic diseases. In this study, we obtained ∼12 million raw reads containing a total of ∼2 million non-redundant tags with high-quality reads. We found that only one-third of these non-redundant small RNA reads demonstrated a perfect match with the draft *S. japonicum* genome, while two-thirds of the reads were mapped to the genome with at least one mismatch. This could be ascribed to the following reasons: (1) genetic polymorphisms. The microRNA data were just matched with published draft *S. japonicum* genome; where the sequences of only assembled contigs, not all, were released into publish domain; (2) systemic errors. The quality of the genomic DNA sequences generated by old DNA sequencing machines could be lower than those generated by new generation DNA sequencer; (3) post-transcriptional edition of miRNA also could contribute to the mismatches observed, which needs to be confirmed.

According to the published criteria for distinguishing bilaterian miRNAs from other types of small RNAs [4], [28], [29], a bioinformatics pipeline (Figure 1) was performed and identified 176 *S. japonicum* miRNAs, including four known *S. japonicum* miRNAs, sja-let-7, sja-miR-71a, sja-miR-125 and sja-bantam [17]. The remaining 172 miRNAs were first recognized and described in *S. japonicum*.

The collection of miRNA data showed that 141 (80%) of the 176 miRNAs were identified in the SC library, while 116 (66%) were identified in the AW library, 81 (46%) of the miRNAs were found in both libraries. Some miRNA genes, including miRNA-2e-5p, miRNA-2e-3p, miR-71b, miR-2a, miR-2f, miR-124 and miR-31, seem to be preferentially enriched in immature worms, whereas miR-125, miR-8 and bantam exhibited a higher abundance in mature adults (Figure 3D). The different miRNAs abundance during different developmental stages of *S. japonicum* implied that some miRNAs could play a distinct role in modulating development, maturation and procreation of the worm, through interfering with the translation and/or mRNA stability of target genes. However, whether the differential display between both libraries is ascribed to the distinct roles of these miRNAs in different life cycle stages is worthy of further investigation.

In addition, 29 miRNA* were found in *S. japonicum* and a few could play functional role due to higher sequencing reads in certain developmental stages. The phenomenon that both a miRNA and a functional miRNA* are derived from a single hairpin precursor could represent a model for the genesis of miRNA genes with novel functionality via the evolutionary processes of subfunctionalization or neofunctionalization [29]. Interestingly, five miRNAs, miR-71b, miR-2a, miRNA-2e-5p, miRNA-2e-3p and miR-2f, were clustered in tandem at the same genomic locus (CNUS0000011792.1), along with the enrichment in immature worms, implying that they might play an important role in this developmental stage by acting in a synergistic manner under the control of the same promoter. More interestingly, other miR-71 and miR-2 family members, miR-71a, miR-2d, miR-2b, and miR-2c, were clustered at another genomic locus (CNUS0000007682.1) in an inverted orientation. It should be pointed out that the cluster of miR-2 family members were found in silkworm (*Bombyx mori*), however, the combination of both miR-2 and miR-71 in the same clusters was not identified in other bilaterian animals.

In addition to these miRNA gene clusters, we also found that both sense and antisense DNA strands within the same genomic loci could generate the distinct miRNAs. The observation that the same genomic loci produce different miRNAs could represent the requirement of ancestral gene for multiple functions [29].

Schistosomes belonging to *Lophotrochozoa* were considered as lower bilaterian animals [37], and thus, the extensive repertoire of *S. japonicum* miRNA genes could provide a unique chance to clarify the evolutionary constraint of *Schistosoma* or *Platyhelminthes* miRNAs, through comparing other bilaterian miRNAs. *Schistosoma* miRNAs with unique genetic characteristics could represent a characteristic procession under selective pressure, or potential ancient prototypes for bilaterian orthologs that would be further evolutionally processed.

These novel types of *S. japonicum* miRNAs could play important roles in development, maturation and host-parasite interplay via modulating the translation and/or mRNA stability of target genes. Although the predictions of miRNA target genes are a very complex process and require experimentation, our prediction could provide some clues for understanding of *S. japonicum* miRNAs functions. For examples, TGF-β receptor II/activin receptor IIA, which plays important roles in schistosomal development including male-induced female reproductive development and host-parasite interaction [38]–[40], could be regulated by 16 miRNAs, including let-7. Some members of cathepsin superfamily involved in hemoglobin proteolysis cascade that may be essential to the mammal-parasitic stages of schistosomes [10], including cathepsins A, B, C, D, K, L and cathepsin B-like cysteine proteinase (Antigen Sj31), could be regulated by several miRNAs (Table S7). Some genes, such as insulin-induced gene 1 protein (INSIG-1), cytosolic thyroid hormone binding protein and thyroid hormone receptor interactor 10, possibly associated with the exploitation of host hormones, could be modulated by *S. japonicum* miR-31 and miR-2d.

In summary, we have created a unique resource, a collection of *S. japonicum* miRNAs that could be used as a new platform to study the genomic structure, gene regulation and networks, evolutionary processes, and developmental features of schistosomes and host-parasite interactions. Although it is still not known how these *Schistosoma* miRNAs are evolutionarily related to each other, the observation that both the miR-71and miR-2 families lie within the same genomic clusters presents a model for the genesis of novel miRNAs, and implies that these genes exert their effects on worm development in a synergistic manner.

## Supporting Information

Figure S1 Phylogenetic analysis of Schistosoma miR-8 and its orthologs or paralogs. Phylogenetic analysis was performed for the known miR-8 and its orthologs or paralogs from bilaterian animals using MEGA 4 software. Abbreviations: sja, *Schistosoma japonicum*; sma, *Schistosoma mansoni*; sme, *Schmidtea mediterranea*; dme, *Drosophila melanogaster*; dre, *Danio rerio*; fru, *Fugu rubripes*; gga, *Gallus gallus*; hsa, *Homo sapiens*; mmu, *Mus musculus*; xla, *Xenopus laevis*; cel, *Caenorhabditis elegans*; csa, *Ciona savignyi*; cin, *Ciona intestinalis*; oan, *Ornithorhynchus anatinus*; xtr, *Xenopus tropicalis*.
(0.36 MB TIF)

Figure S2 Evolutionary analysis of *S. japonicum* miRNAs. Schisitosoma miR-10, miR-124, miR-125, miR-192, miR-31, and let-7 were analysed by comparing all known orthologs from bilaterian animals, excluding schistosomes, as described using WebLogo described above. The nucleotides in red could represent schistosome-specific substitutions that are distinct from all orthologs or their paralogs of other bilaterian animals.
(2.00 MB TIF)

Figure S3 The relationship between Sja-miR-3148 and the host gene heterogeneous nuclear ribonucleoprotein K. Sja-miR-3148 was mapped to the sixth intron of the heterogeneous nuclear ribonucleoprotein K (hnRNP K) gene, which was predicted as one of the potential target genes for sja-miR-3148 by miRanda.
(0.16 MB TIF)

Table S1 The numbers of enzymes and proteins involved in the small-RNA machinery of schistosomes and representative eukaryotes.
(0.03 MB XLS)

Table S2 Genomic mapping distribution of *S. japonicum* non-redundant samll RNA tags from schistosomula (SC) and adult worm (AW) cDNA libraries.
(0.03 MB XLS)

Table S3 List of 176 *Schistosoma japonicum* miRNAs and their associated information.
(0.17 MB XLS)

Table S4 Seed sequences of *S. japonicum* miRNAs and their homology with known miRNAs.
(0.07 MB XLS)

Table S5 Some *S. japonicum* miRNAs with high homology to known miRNAs.
(0.05 MB XLS)

Table S6 31 potential *S. mansoni* miRNAs with high homology to *S. japonicum* orthologs.
(0.03 MB XLS)

Table S7 Predicted target genes of *S. japonicum* miRNAs.
(3.08 MB XLS)

Table S8 The *S. japonicum* miRNAs mapped to the introns of some schistosome genes (host genes).
(0.04 MB XLS)
