# Supplementary figures and images for: Genome-Wide Identification of Schistosoma japonicum MicroRNAs Using a Deep-Sequencing Approach
Source: PLoS One. 2009 Dec 8;4(12):e8206. doi: 10.1371/journal.pone.0008206 (PMC2785426; doi:10.1371/journal.pone.0008206)

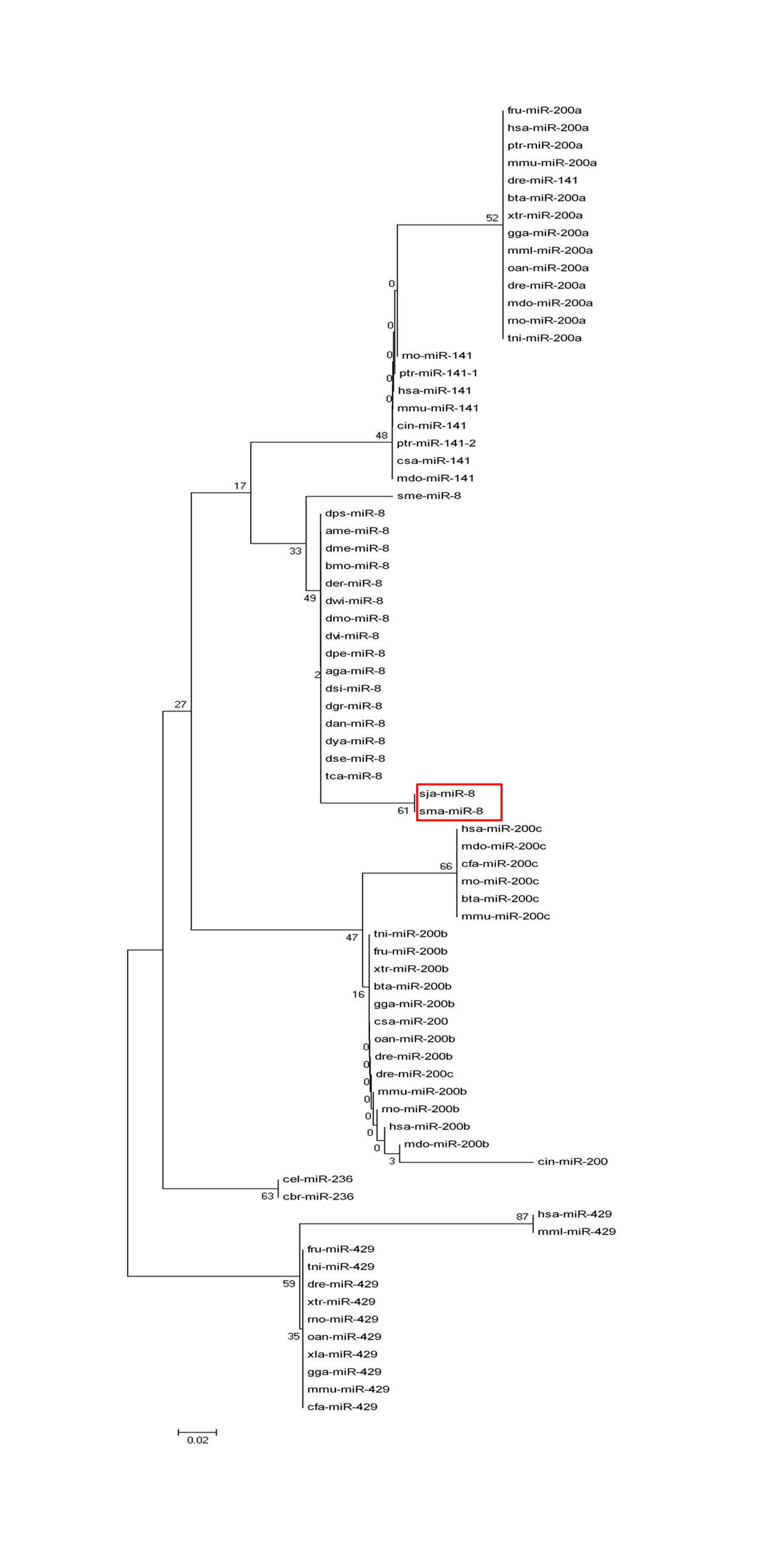

Supplement: Figure S1 — Phylogenetic analysis of Schistosoma miR-8 and its orthologs or paralogs. Phylogenetic analysis was performed for the known miR-8 and its orthologs or paralogs from bilaterian animals using MEGA 4 software. Abbreviations: sja, Schistosoma japonicum; sma, Schistosoma mansoni; sme, Schmidtea mediterranea; dme, Drosophila melanogaster; dre, Danio rerio; fru, Fugu rubripes; gga, Gallus gallus; hsa, Homo sapiens; mmu, Mus musculus; xla, Xenopus laevis; cel, Caenorhabditis elegans; csa, Ciona savignyi; cin, Ciona intestinalis; oan, Ornithorhynchus anatinus; xtr, Xenopus tropicalis. (0.36 MB TIF) [file pone.0008206.s001.tif]

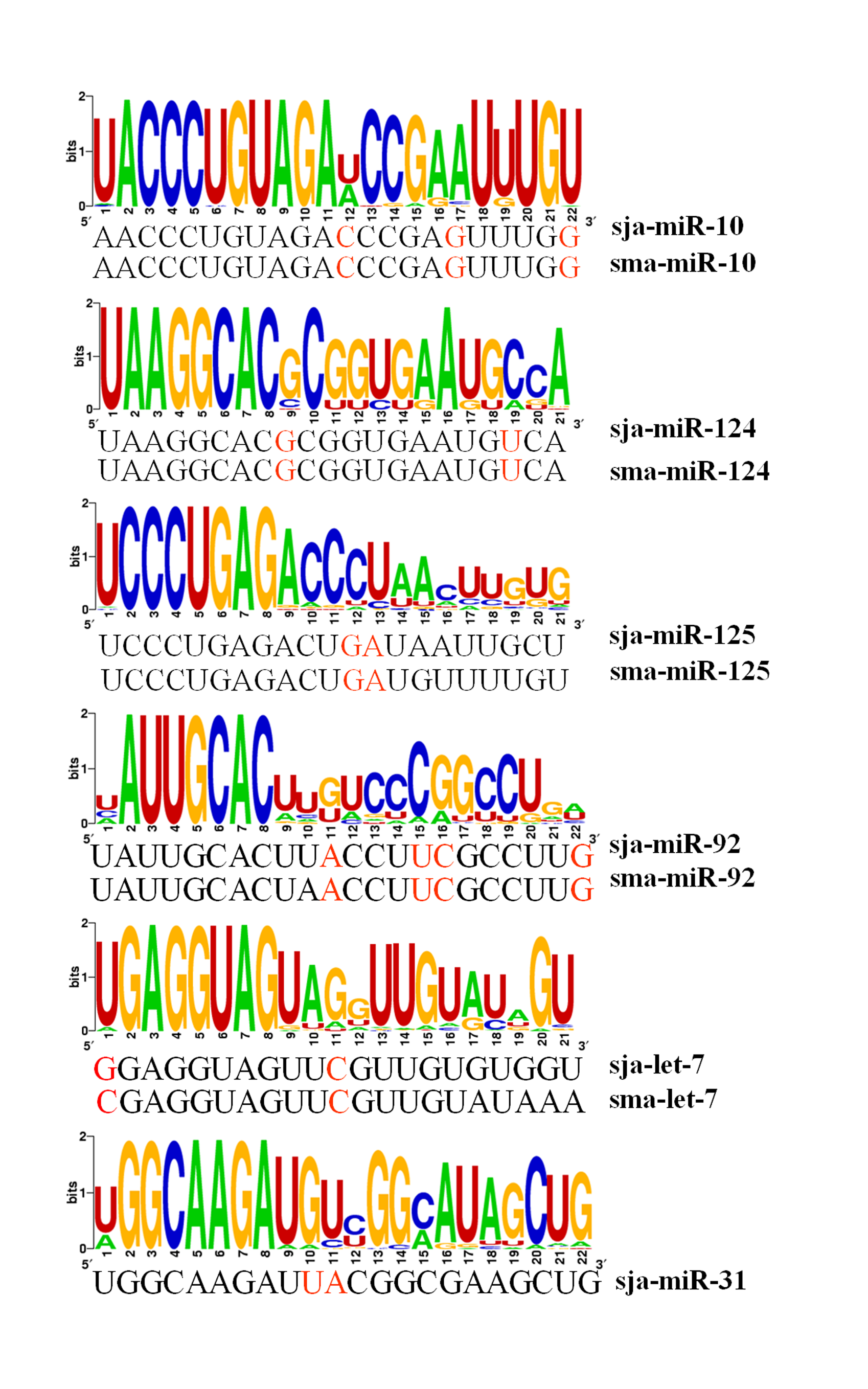

Supplement: Figure S2 — Evolutionary analysis of S. japonicum miRNAs. Schisitosoma miR-10, miR-124, miR-125, miR-192, miR-31, and let-7 were analysed by comparing all known orthologs from bilaterian animals, excluding schistosomes, as described using WebLogo described above. The nucleotides in red could represent schistosome-specific substitutions that are distinct from all orthologs or their paralogs of other bilaterian animals. (2.00 MB TIF) [file pone.0008206.s002.tif]

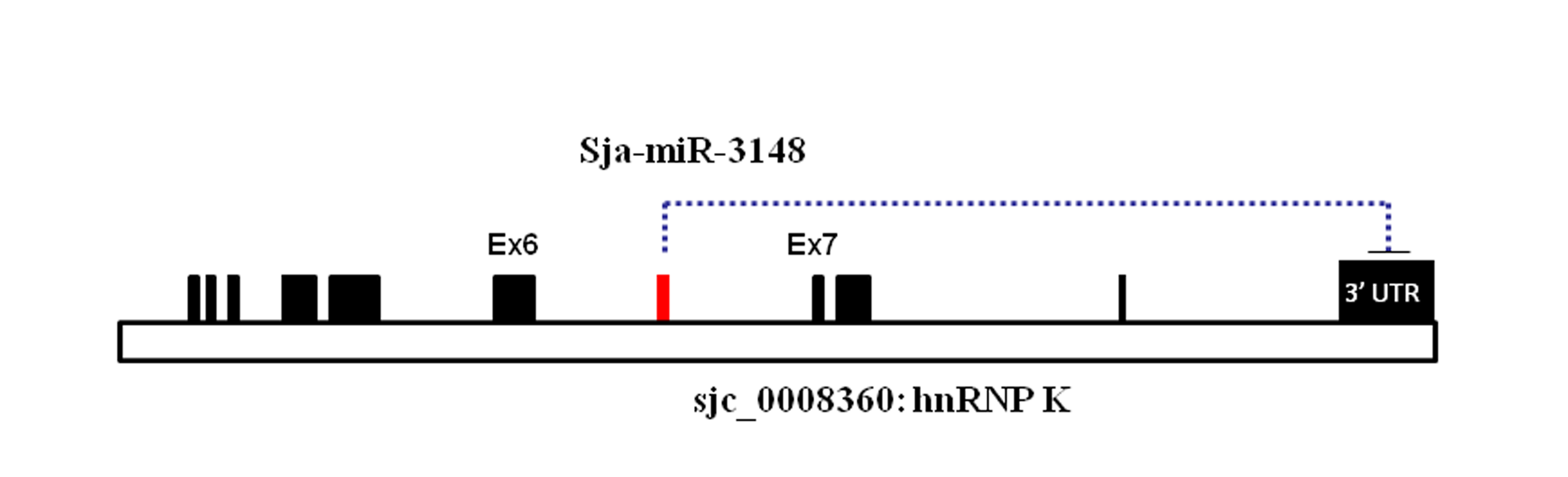

Supplement: Figure S3 — The relationship between Sja-miR-3148 and the host gene heterogeneous nuclear ribonucleoprotein K. Sja-miR-3148 was mapped to the sixth intron of the heterogeneous nuclear ribonucleoprotein K (hnRNP K) gene, which was predicted as one of the potential target genes for sja-miR-3148 by miRanda. (0.16 MB TIF) [file pone.0008206.s003.tif]
